# The Phylodynamic and Spread of the Invasive Asian Malaria Vectors, *Anopheles stephensi*, in Sudan

**DOI:** 10.3390/biology11030409

**Published:** 2022-03-07

**Authors:** Mustafa Abubakr, Hamza Sami, Isam Mahdi, Omnia Altahir, Hanadi Abdelbagi, Nouh Saad Mohamed, Ayman Ahmed

**Affiliations:** 1 Directorate of the Integrated Vector Management (IVM), Federal Ministry of Health, Khartoum 11111, Sudan; elmustafabbkr@gmail.com (M.A.); hamzasani88d@gmail.com (H.S.); isamwahdi@gmail.com (I.M.); 2 Molecular Biology Unit, Sirius Training and Research Centre, Khartoum 11111, Sudan; munno77.fathi@gmail.com (O.A.); hanadi3814@gmail.com (H.A.); nouh_saad@outlook.com (N.S.M.); 3 Institute of Endemic Diseases, University of Khartoum, Khartoum 11111, Sudan; 4 Swiss Tropical and Public Health Institute (Swiss TPH), 4123 Allschwil, Switzerland; 5 Faculty of Science, University of Basel, Petersplatz 1, 4001 Basel, Switzerland

**Keywords:** *Anopheles stephensi*, invasive disease vector, phylogenetic analysis, haplotypes analysis, malaria epidemics, International Health Regulations, vector control and surveillance, climate change, Africa

## Abstract

**Simple Summary:**

Climate change increases the suitability of some environments for the establishment of newly introduced insects and is a major driver for the spread of mosquitoes that transmit diseases. *Anopheles stephensi* is a disease vector mosquito that transmits malaria and is naturally endemic in Asia. This vector newly emerged in Africa (first report from Djibouti in 2012), where annual malaria infections and deaths are the highest worldwide. This mosquito has different ecology and behavior from previously known malaria vectors in Africa, which makes control difficult for local under-resourced health systems. Considering the capacity of this vector to transmit at least two malaria-causing parasites (*Plasmodium falciparum* and *Plasmodium vivax*), we investigated its distribution and population structure in Sudan and assessed the potential risk of its further spread into neighboring countries. Using morphological and genomic sequencing techniques, we confirmed the presence of *Anopheles stephensi* along the borders of six countries previously assumed as free, including Chad, Egypt, Eritrea, Libya, Republic of Central Africa, and South Sudan. African countries need to enhance vector surveillance and control services and utilize genomics tools for tracking the dynamics of invasive disease vectors.

**Abstract:**

*Anopheles stephensi* is an invasive Asian malaria vector that initially emerged in Africa in 2012 and was reported in Sudan in 2019. We investigated the distribution and population structure of *An. stephensi* throughout Sudan by using sequencing and molecular tools. We confirmed the presence of *An. stephensi* in eight border-states, identifying both natural and human-made breeding sites. Our analysis revealed the presence of 20 haplotypes with different distributions per state. This study revealed a countrywide spread of *An. stephensi* in Sudan, with confirmed presence in borders states with Chad, Egypt, Eritrea, Ethiopia, Libya, Republic of Central Africa, and South Sudan. Detection of *An. stephensi* at points of entry with these countries, particularly Chad, Libya, and South Sudan, indicates the rapid previously undetected spread of this invasive vector. Our phylogenetic and haplotype analysis suggested local establishment and evolutionary adaptation of the vector to different ecological and environmental conditions in Sudan. Urgent engagement of the global community is essential to control and prevent further spread into Africa.

## 1. Introduction

Malaria is a serious life-threatening illness that is a public health risk for humans mainly in rural tropical areas worldwide. In 2019, up to 252 million malaria cases and 460,000 related deaths were estimated globally [1]. Africa suffers the highest burden of malaria, with over 94% of malaria cases and related deaths reported from African countries [1]. This proportionally high risk of malaria in Africa is attributed to the existence of several competent vectors, including different species of *An. gambiae* and *An. funestus* complexes [2,3]. More importantly, pregnant women and children under 5 years of age are the most vulnerable groups affected by the disease, with around 67% malaria reported deaths among children under the age of 5 years [1]. Malaria control programs worldwide rely on two approaches for the prevention and control of the disease: chemotherapy for case management and insecticide-based vector control tools (insecticide-treated bed nets (ITNs), and indoor residual spraying (IRS)) [4]. However, both approaches are challenged by rapidly growing resistance to antimalaria drugs [5] and insecticide resistance among disease vectors [6]. The recently approved malaria vaccine might offer a better alternative for protecting people at high risk of malaria infection [7]. The recent World Malaria Report 2021 highlighted an increase in malaria cases (14 million) and deaths (47,000), mainly attributed to the ongoing COVID-19 pandemic [8].

Malaria is an important threat to public health throughout Sudan, with 1% of the world malaria cases and deaths reported there [2]. According to the 2019 World Malaria Report, around 4.4 million malaria cases were estimated in Sudan, contributing more than 45% of the total number of cases reported in the World Health Organization (WHO) Eastern Mediterranean Region (EMRO). About 13,000 malaria-related deaths were estimated in the country [2]. Unfortunately, morbidity and mortality of malaria in Sudan increased by nearly 50% between 2015 and 2019 [2]. Although the country’s entire population lives with the risk of malaria, endemicity and burden are heterogeneously distributed [9]. This variation in malaria transmission can be attributed to several risk factors, including varied distribution and composition of competent vectors of malaria per different State [3,10], ecology, environment, climate change [3,11,12], conflicts, and human population displacement [9,11]. The composition of malaria vectors in Sudan includes different members of the *An. gambiae* and *An. funestus* complexes, *An. pharoensis*, and *An. Arabiensis* [2,3,10]. The distribution and spread of disease and disease vectors could be significantly influenced by climate change [12].

Recent emergence of the malaria vector *An. stephensi* in Sudan carries a serious threat of malaria epidemics and may be an additional risk factor contributing to the current increase in malaria morbidity and mortality in Sudan [9,13,14,15]. Emergence of this invasive malaria vector in Djibouti was associated with an epidemic [16,17]. After initial reports on emergence of *An. stephensi* in Sudan in 2019 [14,15], unusual increases in malaria cases were reported in different regions, suggesting the involvement of *An. stephensi*, similarly to Djibouti [1,2,9]. This study was prompted by the alarming WHO alert encouraging countries in the Horn of African to enhance their vector surveillance to delineate the spread of this invasive malaria vector in the region [18]. We report our findings related to the spread of *An. stephensi* in Sudan and its presence and absence in regional representative States.

## 2. Materials and Methods

### 2.1. Mosquito Collection

We followed two strategies to collect mosquito samples, (1) active surveys targeting *An. stephensi* mosquitoes and (2) routine vector surveillance, which aim to collect and detect vectors for all diseases. To collect *Anopheles* mosquitoes, we used aspirators and BG-Sentinel traps for adults and dippers for aquatic stages (larvae and pupae).

### 2.2. Active Surveys Targeting An. stephensi

**Figure 1:**
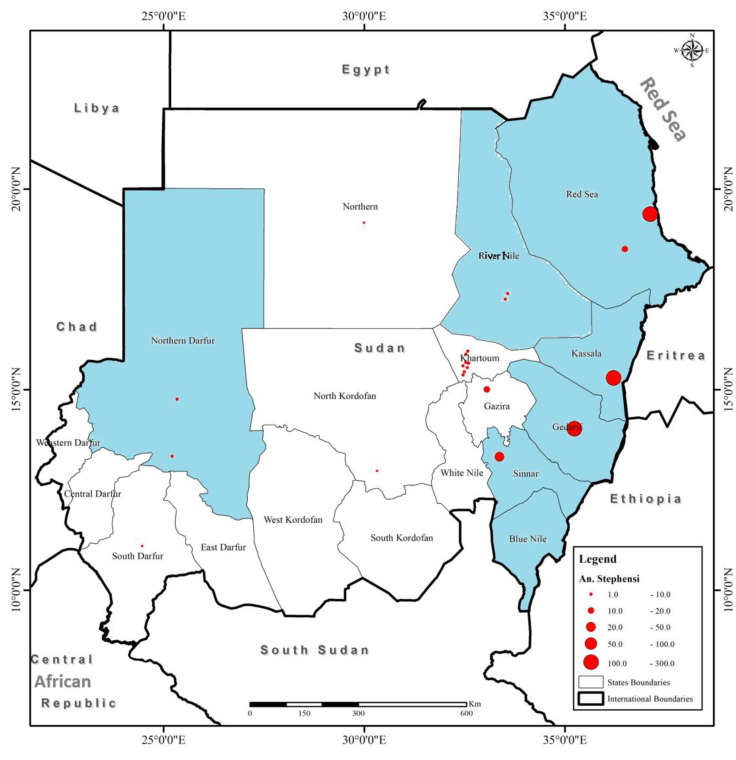
Sudanese States (highlighted in light blue) where active surveys were implemented to detect *An. stephensi* mosquitoes. The red circles indicate the number of *An. stephensi* mosquitoes collected.

We implemented active *An. stephensi*-targeted surveys in States considered at high risks for emergence. The risk of *An. stephensi* emergence in each State was assessed based on three factors: (a) previous reports about vector presence in the State (Gedaref, Kassala, and the Red Sea States), (b) sharing a border with a State or country with confirmed vector presence (the Blue Nile and Sennar states), and (c) unusual increases in malaria cases (North Darfur State) (Figure 1).

### 2.3. Routine Vector Surveillance

In addition to active surveys, the national department of Integrated Vector Management (IVM) conducted a series of extensive trainings for national public health officers on collection, identification, and control of disease vectors throughout the 18 States. Routine vector surveillance operating throughout the country captured the presence of *An. stephensi* in Sennar, Gezira, Khartoum, and River Nile States.

### 2.4. The Morphological Identification of Mosquito Samples

We transported the mosquito larvae that we have collected to the insectary at the National Public Health laboratory where we reared them to adults. We have used the standard morphological keys for Afrotropical *Anopheles* mosquitoes for the morphological identification of the mosquito samples to species level [19].

### 2.5. DNA Extraction from Mosquito

Following manufacturer instructions, we extracted the total DNA from the mosquito samples using QiaAmp tissue extraction kits (Qiagen, Hilden, Germany). We checked the quality of the DNA using a nanodrop spectrophotometer (ND1000, Houston, TX, USA) following the manufacturer’ guidelines and then preserved them at −20 °C until molecular examination.

### 2.6. Polymerase Chain Reaction (PCR)

We have used Folmer primers (LCO1490 and HCO2198) to amplify the cytochrome oxidase 1 (CO1) region of the mitochondrial DNA of the mosquito genomes in a thermocycler PCR machine (Applied Biosystems, ThermoFisher Scientific, Budapest, Hungary) [20]. We have run PCR by taking 2 µL of the extracted DNA and adding to a 4 µL PCR master mix (Solis Biodyne, Tartu, Estonia), which consisted of 1 U DNA polymerase, 12.5 mM MgCl2, and 4 mM dNTPs. PCR cycling conditions were as follows: initial denaturation at 95 °C for 5 min, 35 cycles of denaturation at 95 °C for 30 s, annealing at 58 °C for 30 s, and extension at 72 °C for 30 s, with a final extension step of 72 °C for 10 min. Following amplification, we visualized PCR products on gel electrophoresis (Major Sciences, Saratoga, CA, USA.) by loading PCR products on 2% agarose gel and placed them onto 150 V and 75 A for 1 h. The amplified PCR products were sequenced after being checked for band quality, including band sharpness and thickness intensity under UV-light using the Sanger Deoxyribonucleic acid sequencing method by 3730XL DNA analyzer (Applied Biosystems, Waltham, MA, USA) through Macrogen company (Macrogen Inc, Amsterdam, The Netherlands).

### 2.7. Sequences’ Identity Confirmation

We have checked the identity of our sequences of the cytochrome c oxidase 1 region of the mitochondrial DNA by comparing their similarity with the worldwide-published sequences of *An. stephensi,* using the online BLAST nucleotide algorithm available in the NCBI GenBank database (https://blast.ncbi.nlm.nih.gov/Blast.cgi, accessed on 1 October 2021). We have deposited our sequences of *An. stephensi* that we have obtained during this study into the GenBank database at NCBI under accession numbers OK216338 to OK216737.

### 2.8. Bioinformatics Analysis

We have used GENtle software (v1.9.4) to check the correctness of sequencing peaks and nucleotides base-calling errors that could occur during sequencing [21]. We trimmed the primer’s sequences to reduce sequencing mismatching that occurs at the start of the sequencing process [22]. We analysed the sequences to identify novel cox1 gene sequence polymorphism using MEGA7 software by aligning the sequences with the reference sequence KT899888.1; it belongs to the An. stephensi strain Hor that was originally collected from India and reared in the Third Military Medical University, China [22,23]. We considered the nucleotide substitution model with the lowest Bayesian Information Criterion (BIC) scores as the best-fit model; Tamura 3-parameter model [24]. We have modelled the non-uniformity of evolutionary rates among sites using a discrete Gamma distribution for creating the phylogenetic tree [24]. We have obtained the sequence diversity parameters including the number of haplotypes (Hap), haplotypes diversity (Hapd), segregating sites (S), and average number of nucleotide differences between two sequences (Pi) using the software DnaSP v5.10 [25]. We have constructed haplotypes’ network by developing the median-joining network using popART software (v4.8) (http://popart.otago.ac.nz, accessed on 1 October 2021). To test the natural selection theory and the population expansion of *An. stephensi*, we performed the FuFs statistics and Tajima’s D tests. We have estimated the degree of the *An. stephensi* genetic diversity by comparing our sequences to previously published sequences and calculated the pairwise fixation index (Fst) using DnaSP [25].

## 3. Results

Our findings confirm the widespread invasive malaria vector, *An. Stephensi*, in Sudan. Additionally, we identified 20 haplotypes among the *An. stephensi* populations that we have collected from different states (Figure 1).

### 3.1. Surveillance Data

We collected mosquito samples by using active surveys from six States distributed throughout Sudan, namely Gedaref, Kassala, Red Sea, River Nile, North Darfur, and Sennar States. We preserved and shipped the collected samples to Khartoum, where we have identified the presence of *An. stephensi* morphologically and molecularly. We confirmed the identity of our sequence by performing a BLAST search on the NCBI database. We have collected four different species of *Anopheles* mosquitoes from the different states, namely *An. arabiensis*, *An. macupalpis*, *An. rufipes*, and *An. stephensi* (Table 1). However, the proportion of *An. stephensi* from the total collected *Anopheles* mosquito samples differs per State, with the highest percentage in the Red Sea States (100%), 94% in Gedarif, and only 2% in North Darfur. We did not detect *An. stephensi* in the Blue Nile State (Table 1) and (Figure 1). Additionally, the presence of *An. stephensi* was later confirmed through routine surveillance in four new States, including Gezira (1 sample), Khartoum (five samples), Northern (three samples), North Kordofan (one sample), and South Darfur (seven samples) (Figure 1).

### 3.2. Breeding Sites of An. stephensi

**Figure 2:**
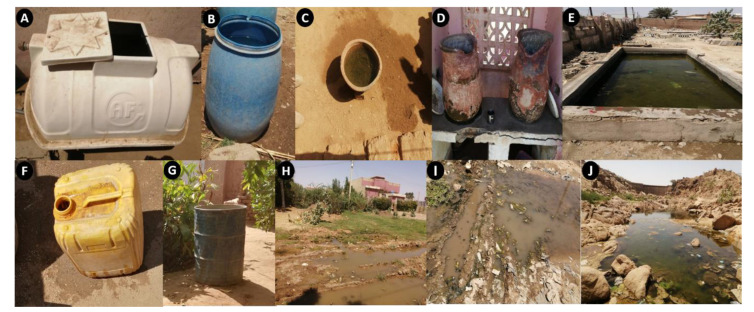
Representative breeding sites that were positive for presence of aquatic stages of *An. stephensi* (larvae and pupae). (**A**) Plastic cistern, (**B**) plastic barrel, (**C**) mud pot, (**D**) clay pot (Zeir/Jar), (**E**) ground water-basin, (**F**) plastic jerrycan, (**G**) iron barrel, (**H**,**I**) leakage of broken water supply, and (**J**) rainwater pond trapped in a rocky valley.

Several different types of breeding sites were positive for the presence of *An. stephensi* during the larvae collection, as shown in Figure 2. Cement, clay, and plastic containers were the most common and productive containers in the eastern region, while iron containers (e.g., barrels) were the most productive containers in the western region of the country (Figure 2).

### 3.3. Bioinformatic Analysis

#### 3.3.1. Haplotype Analysis

**Figure 3:**
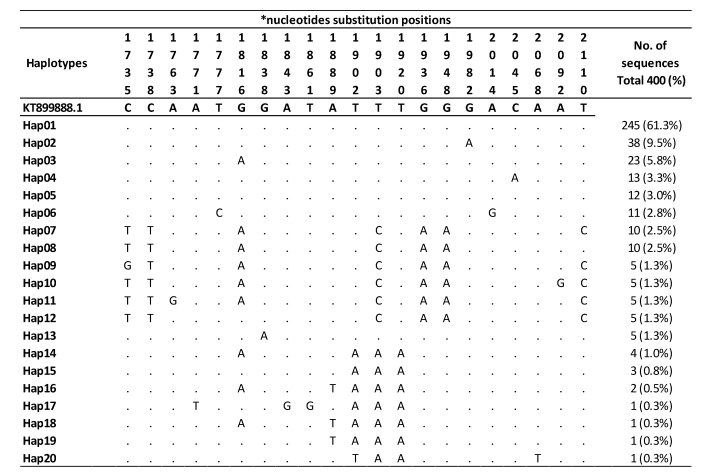
Sequence alignment of the 20 Sudanese *An. stephensi* haplotypes. Substitutions were indicted with their nucleotide codes; no deletion nor insertions were present. The dots (.) indicate identical nucleotides at the specified position in comparison with the reference sequence KT899888.1 (ref). * Nucleotide substitution positions, based on the start of the complete mitochondrial cytochrome c oxidase 1 (CO1) gene, are read vertically.

Results of the sequence alignment compared to the reference sequence KT899888.1 showed the presence of different nucleotides polymorphisms at different sites in the CO1 gene. However, all detected polymorphisms and nucleotides substitutions were nonsynonymous substitutions (Figure 3). We have identified 20 haplotypes (named as Hap 01-20) among the collected Sudanese populations of *An. Stephensi,* with a Hapd of 0.6091 ± 0.00077. The number of segregating sites (S) detected was 21, and the average number of nucleotide differences between two sequences (Pi) was 0.00486. The most frequent Hap detected was Hap01, which was detected among 245 (61.3%) samples, followed by Hap02 at 38 (9.5%) and Hap03 at 23 (5.8%). The remaining haplotypes constituted a low frequency (Figure 3).

#### 3.3.2. Phylogenetic Analysis

**Figure 4:**
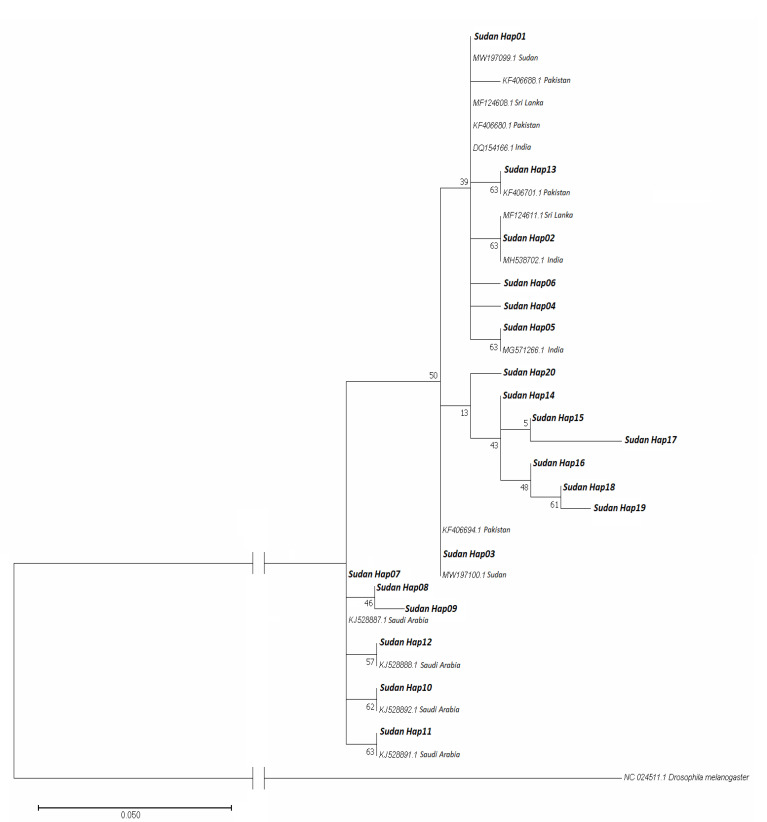
Phylogenetic tree showing the relationship between the Sudanese *An. stephensi* haplotypes with 15 reference sequences. Sudanese haplotypes (Sudan Hap01–Sudan Hap20) are in bold. The reference sequences along with their accession numbers and origin of isolate were included for each. Drosophila melanogaster was used as an outgroup taxon.

We have constructed the phylogenetic tree based on the Tamura 3-parameter model by adding reference sequences to investigate sequence relations based on origins. Sequences added for the analysis are presented in Supplementary Files (Table S1). The phylogenetic tree showed that Hap01, 02, 04, 05, 06, and 13 were all clustered with sequences reported from Pakistan, Sri Lanka, India, and the previously deposited sequences from Sudan. Hap03 clustered with sequences from Pakistan and Sudan. Hap14, 15, 16, 17, 18, and 19 were clustered separately, while Hap07, 08, 09, 10, 11, and 12 were clustered with sequences reported only from Saudi Arabia (Figure 4)

#### 3.3.3. Worldwide Haplotypes Analysis

We trimmed the ends of the obtained sequences to standardize the length of sequences before performing sequence alignment, which has resulted in 19 different haplotypes. The Hapd of the worldwide sequences was 0.5850 ± 0.00064. The average number of pairwise nucleotide differences was 1.338, and nucleotide diversity was 0.00385. Further, we grouped sequences based on origin and analyzed them to investigate genetic diversity and the natural selection theory (Table 2). Countries with the highest number of haplotypes detected were Pakistan and Saudi Arabia, with four haplotypes each. However, study sequences showed a high number of haplotypes among the Red Sea, Kassal, and Al Gedarif states, with 11 Haps. Their Hapd was 0.539 ± 0.00169, 0.579 ± 0.00339, and 0.64 ± 0.00279, respectively. Although we have identified 11 different haplotypes, only a few diversities were present, indicating that the population in these three states is partially conserved or their divergence has started recently. However, we observed the complete divergence between the haplotypes for haplotypes present in North Darfur state where Hapd was 1.0 ± 0.00926. The natural selection theory tests, Tajima D, and FuFs tests revealed that the only significance of the FuFs test was for Pakistan (Table 2). For most of the analyzed sequences, Tajima D and FuFs tests were negatively insignificant (*p* value > 0.05) in North Darfur, Red Sea, Kassala, and Gedarif (Table 2).

We computed the pairwise Fst test to estimate the degree of gene flow among the different populations. The values of Fst test among the populations ranged from 0.004 to 0.927 and were all statistically significant (*p* value < 0.05) (Table S2. in Supplementary Files). Values of Fst test were not very high between Sudanese *An. stephensi* sequences. A high value of Fst test indicates that most of the populations were genetically differentiated (Table 3).

We clustered all sequences belonging to *An. stephensi* from Sudan in a single population to reduce the bias in population genetic differentiation. The values of Fst test for the genetic differentiation were also statistically significant, *p* value < 0.05 (Table S3 in Supplementary Files).

**Figure 5:**
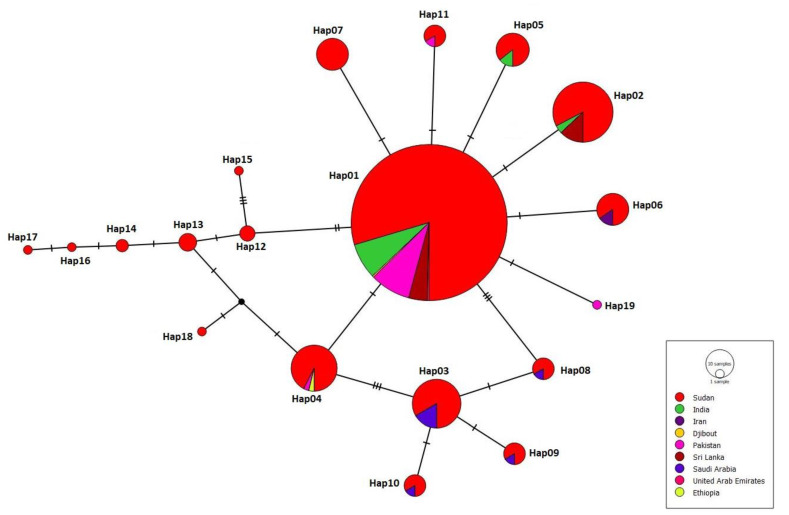
Median-joining haplotype network of the 19 Sudanese *An. stephensi* haplotypes and worldwide *An. stephensi* sequences. Haplotypes of each region are presented in color code. Black dashes between the haplotype lines represent the number of substitutions.

The constructed haplotype network shows the existence of a shared haplotype, Hap01 (Figure 5). Hap01 was a major haplotype in all the populations and was consistently placed in the center of the haplotype networks. Only sequences from Saudi Arabia, Iran, and Ethiopia did not include Hap01 (Table S4 in Supplementary Files). The haplotype network exhibited a typical star-like expansion from the main founder Hap01, with the presence of several unique haplotypes presented in certain populations (Figure 5).

**Figure 6:**
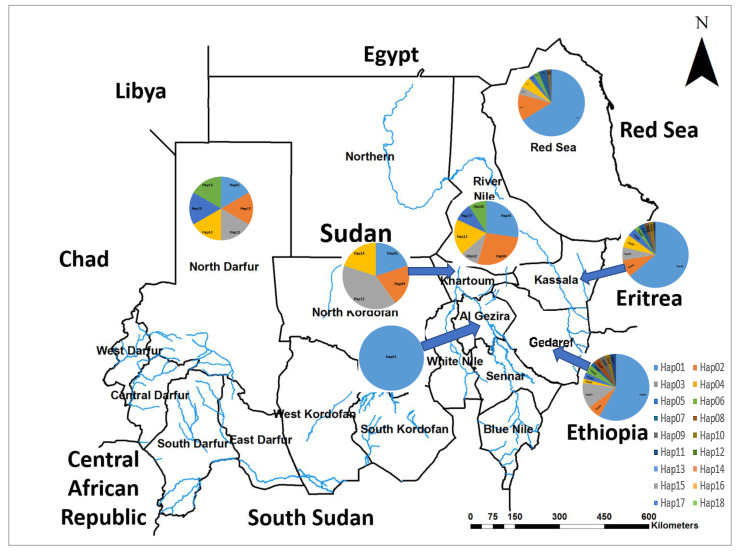
Pie charts inserted to show the haplotypic composition of *An. stephensi* population across the states of Sudan. Haplotypes of each region are presented in color code.

Analyzing the distribution of the different haplotypes that we have identified in this study revealed the predominant distribution of Hap01 throughout most of the country. Hap17 and Hap18 were confined to the River Nile state; meanwhile, Hap15 and Hap16 were confined to the North Darfur state (Figure 6).

In Supplementary Files Table S4, we show the distribution of the different haplotypes among different regions.

## 4. Discussion

We report the large spread of the invasive malaria vector *An. stephensi* in Sudan and the high haplotype diversity, indicating vector local establishment and adaptation to the different environments in the country (Figure 6). The emergence and spread of *An. stephensi* in Africa, including Sudan, is of global importance because it threatens regional public health [14,15,16,26]. This was further indicated by the prompt release of vector alert by WHO in 2019 [18]. The spread of this invasive vector carries the threat of malaria epidemics in urban settings of Africa, particularly in densely populated cities such as Khartoum, Kadugli, and Al Fashir [9,13,14]. Documentation of emergence of *An. stephensi* in Africa is recent, first in Djibouti in 2012 [16] and then in Ethiopia in 2016 [26].

Since the first report of the emergence in country in 2019 [14,15], widespread *An. stephensi* across Sudan was suggested by epidemiological reports that highlighted recent increases in malaria cases and epidemics in some regions, particularly in the southeast and southwest [9,11]. This is similar to the unusual increase in malaria cases in Djibouti associated with the first emergence of *An. stephensi* [16,17].

Climate change is a major driver for the spread of invasive disease vectors as it increases suitability of new environments for novel vectors to establish and eventually accelerate the invasion [27,28]. The influence of climate change on the emergence and distribution of vector-borne diseases and their outbreaks in Sudan is rapidly growing [29]. This is further underscored by the emergence of several arboviruses and their vectors including dengue, Crimean–Congo Hemorrhagic Fever (CCHF), Chikungunya, and Rift Valley fever in different regions of the country [11,12,30,31,32,33,34]. Therefore, the current rapid spread of *An. stephensi* throughout Sudan could be attributed to climate change. In particular, we report the presence of this invasive vector in areas located at more than 1000 km from the nearest point that is predicted to be environmentally suitable for the spread of *An. stephensi* [35]. More importantly, local and international studies have reported that climate change is a major driver for the increase in the transmission of malaria and other vector-borne diseases [36,37]. As a sub-Saharan country where most of the environment throughout the country constitutes deserts and semi-deserts, the survival of diseases vectors is mainly limited to microenvironments surrounding water bodies with grassy covers such as banks of rivers [3]. In such environments, the transmission of vector-borne diseases including malaria is climate dependent, with factors such as rainfall, maximum temperature, relative humidity, and the level of river and flooding having direct and significant influences on malaria transmission and epidemiology [36]. Unfortunately, during recent years, Sudan has suffered from extreme weather events, including heavy rains, severe flooding, and rainstorms, throughout the country [12]. The direct impacts of these climate change phenomena on malaria transmission include but are not limited to the substantial increase in suitable breeding sites, increasing the environmental suitability for vector survival beyond the parasite’s incubation period inside the vectors (the extrinsic incubation period) long enough to breed [38,39,40,41]. Furthermore, heavy rains and flooding are hindering public health services and interventions by limiting accessibility to implementation sites and direct interfering with interventions, such as washing out insecticides [41]. This is particularly alarming when reviewed with our finding of *An. stephensi* aquatic stages (larvae and pupae) in rainwater ponds on the ground, confirming the adaptability of this vector to locally available environments (Figure 2).

Studies in Djibouti and Ethiopia indicated the role of *An. stephensi* in changing malaria transmission and epidemiology in the region, which is further underscored by several malaria epidemics in the area [9,13,17,18]. The risk of malaria epidemics in the urban settings in Africa is predicted if *An. Stephensi* spread into these settings [13,42]. However, several epidemics of malaria have occurred in Sudan in association with extreme weather events, but limited or no entomological investigations were carried out. An epidemic of malaria occurred in the Gezira state in 2013 following heavy rains and flash flood [38]. In correspondence with unprecedented heavy rains and flooding throughout Sudan, malaria cases in the country reached the epidemic threshold; however, the majority of cases were reported among the fragile populations living in humanitarian areas in the war-torn regions of Kordofan and Darfur [9,13,43]. Over 110, 103, and 45 thousand cases of malaria were reported from South, North, and East Darfur states, respectively [43].

Field reports and experimental studies confirmed the competency of *An. stephensi* populations in Africa to transmit both *Plasmodium falciparum* and *P. vivax* [18,44,45]. However, little is known about the dynamics and routes of vector introduction in Sudan and other countries in the Horn of Africa [14,15], although a recent study has suggested that *An. stephensi* in Sudan was introduced from Ethiopia [15]. However, Hap01 has never been reported from Ethiopia, yet it is the major haplotype in Sudan from which other haplotypes might have originated. This suggests that *An. stephensi* in Sudan has not been introduced from Saudi Arabia, Iran, nor Ethiopia due to the lack of Hap01 in their populations of *An. stephensi* (Table S4). Our median-joining haplotype network indicates that *An. stephensi* in Sudan was most likely introduced from countries that share the common haplotype Hap01, namely Pakistan, India, and Sri Lanka (Figure 5). However, we exclude Sri Lanka as a potential source for the origin of this vector because it has been reported there in 2017 as an invasive vector rather than endemic, while evidence confirmed its presence in Sudan since 2016 [15,44]. The extreme gaps in knowledge about bionomics, actual distribution, and susceptibility of *An. stephensi* populations with respect to locally available vector control tools in Africa urge the need for the implementation of research to generate evidence from the field in order to guide intervention and inform policymakers [14,15,16,42]. High coordination and timely data sharing are essential for improving the implementation of vector control strategies and response plans [45]. It is critical to enhance the implementation of International Health Regulations (IHRs 2005), particularly at points of entry/exit between current *An. stephensi* free and already invaded areas [18,45]. Considering the large size of the country and the borders with seven countries, including Chad, Central African Republic, Egypt, Eritrea, Ethiopia, Libya, and South Sudan, the current spread of *An. stephensi* in the country is very alarming nationally and regionally. We confirm the presence of *An. stephensi* in eight States with open international borders (Figure 1). The local establishment of *An. stephensi* in Sudan is further highlighted by the high haplotypic variation (20 haplotypes) detected among the collected populations of *An. stephensi* (Figure 3, Figure 4 and Figure 5).

Previous studies suggested that *An. stephensi* was only breeding in artificial water containers in African countries [46,47]. Our study also found *An. stephensi* larvae in ground breeding sites resulting from leaking water supply pipelines, in agreement with reports from Iran [48]. Variations in the nature of the breeding sites might be attributed to vector adaptation to local ecological, climate, and environmental conditions. Similar preference variation in breeding sites was observed for *Aedes aegypti*. In East Sudan, *Ae. aegypti* larvae were commonly found in high density in clay and cement containers with lower densities in iron containers, while the reverse was found for *Ae. aegypti* in West Sudan (Ahmed et al., unpublished data). This could be attributed to the fact that during the rainy/transmission season in West Sudan, the weather is relatively colder. Heat-absorbent iron containers with warmer water might be more attractive for egg-laying female mosquitoes (influencing breeding site selection) or might increase the hatchability of eggs and eventually the productivity of the breeding site [49,50]. Such an adaptation to on-ground breeding could be how the spread of *An. stephensi* in Sudan is influenced by climate change, specifically heavy rain and flooding [12,27]. In our study, on-ground concrete tanks for water storage were the most productive breeding-site (Figure 3).

Recent malaria epidemics have occurred in areas of Sudan with relatively high coverage of the vector control tools, indoor residual house spraying, and long-lasting insecticidal nets [4,9,13]. The biological and/or behavioral resistance of *An. stephensi* populations in Africa to the currently used insecticides was reported by WHO and recent studies [8,51]. Therefore, the Ministries of Health in Africa need to restructure their vector surveillance and control systems to become more capable in preventing, detecting, and controlling diseases vectors and proactively response to any change in their species composition [52]. The national malaria control programmes in the area should modify their vector surveillance systems to be more vigilant for the emergence and spread of invasive disease vectors and include house inspection for vectors breeding in human-made water containers into their package of routine surveillance [42,53]. Vector surveillance systems should incorporate genomics sequencing and molecular tools into their vector identification protocol to avoid former delay in the detection of novel vectors due to morphological similarity [15,42]. While IVM programmes could implement larvae source management to control *An. stephensi* and the co-existing *Ae. Aegypti*, however, the co-effectiveness of this approach should be systematically evaluated in the field. Governments and local and international partners of the health cluster including WHO and Global Fund should support the implementation of more cost-effective and environmentally friendly (non-insecticide based) vector control interventions [52]. Particular attention should be provided for protecting women, children under five years old, and poor and fragile communities, especially those living in humanitarian settings [9,12,13,52].

As part of the action plan and response strategy to the emergence of *An. stephensi* in Sudan (Vector Control Strategic and Response Plan for Sudan 2021–2025), the IVM department and the Federal Ministry of Health have mobilized their local resources to implement active vector surveillance to confirm the presence or absence of *An. stephensi* in specific States of high risk (The Red Sea, Kassala, Gedaref, River Nile, Sinnar, and North Darfur States) (Figure 1). This risk assessment was carried out based on vector presence in/nearby the specific area and is based on the epidemiological recording of malaria cases (Sudan Strategic Plan for the Surveillance and Control of *An. stephensi* in Sudan, 2021–2025).

Interestingly, all areas where active surveys were implemented were positive for the presence of *An. Stephensi,* except for the Blue Nile State (Figure 1). In parallel, the IVM department invested in expanding vector surveillance and control by training national surveillance officers on the surveillance and control of different disease vectors instead of only focusing on *An. arabiensis*. Unfortunately, it seems that the previous lack of implementing integrated vector surveillance allowed this vector to spread throughout the country undetected and become established [14,15].

Alarmingly, an experimental study has shown that the infection of *An. stephensi* with malaria parasites facilitate Rift Valley fever viruses [54]. This could justify the recently reported changes and emergence events of Rift Valley fever in Sudan [30,33,34]. Similarly, another independent experimental study revealed the capacity of *An. stephensi* to transmit Chikungunya virus [55], and this is further supported by the correspondence of the massive outbreak of Chikungunya in Sudan in the areas heavily manifested by *An. stephensi* [30]. More importantly, if *An. stephensi* was confirmed to transmit the Chikungunya virus in field conditions, the recent large outbreak of Chikungunya (27,540 cases reported) might be an indicator for the spread of this vector into Chad [56]. Therefore, entomological investigations are essential during outbreaks of arboviral diseases in the area to determine the role of *An. stephensi* in the transmission of these deadly and economically devastating diseases.

Although vector-borne diseases constitute a major threat to public health in Sudan, surveillance and control of disease vector services in the country are limited by several persistent issues, including poverty, poor sanitation, unplanned urbanization, the underfunded and weak health system, and the high turnover of trained personnel [1,53]. Furthermore, limited resources, relatively high rate of conflicts, humanitarian crisis, massive human population displacement, and climate change are the major drivers for the emergence and outbreaks of vector-borne diseases in Sudan [11,30,31,32,33,34].

## 5. Conclusions

The invasive malaria vector *An. stephensi* is well spread throughout Sudan and has potentially been introduced into neighboring assumedly *An. Stephensi*-free countries already. The population structure, haplotypic variation, and change in breeding site preferences of *An. stephensi* in Sudan indicate adaptation and establishment and suggest that the invasion occurred earlier than 2016, possibly through multiple introductions most likely from Pakistan and/or India. The rapid expansion of the geographical distribution of this vector emphasizes the need for global engagement with all relevant stakeholders, including donors and technical supporters such as different UN agencies, national programs, and the Ministry of Health to invest early in the control and elimination of this invasive vector before it fully adapts and become endemic. Furthermore, countries in Central and West Africa and their health partners should be alarmed by the potential risk of introducing this invasive vector into their areas, which further necessitates strict implementation of IHRs to control disease vectors, particularly at points of entry/exit. Additionally, vector surveillance and control systems in Africa should shift from doing business as usual surveillance to be more vigilant for the spread of invasive diseases vectors through the incorporation of genomics and molecular tools into their vector surveillance. Moreover, health stakeholders, mainly WHO and Global Fund, should invest more and support the implementation of the environmentally friendly non-insecticides-based vector control interventions such as larvae source management, which might be a more cost-effective tool for integrated vector management.

## Figures and Tables

**Table 1:** Identity and percentage of *Anopheles* mosquito samples collected by conducting active entomological surveys from different States of Sudan in 2021.

No.	State	*An. stephensi*	*An. arabiensis*	*An. macupalpis*	*An. rufipes*	% of *An. stephensi*	Total
1	Blue Nile	0	124	0	0	-	124
2	Gedarif	163	10	0	0	94.2%	173
3	Kassala	106	80	0	0	57.1%	186
4	North Darfur	6	219	60	0	2.1%	285
5	Red Sea	309	0	0	0	100%	309
6	River Nile	11	254	0	0	4.2%	265
7	Sinnar	29	232	0	25	10.1%	286
	Total	630	921	60	25		1628

**Table 2:** Diversity and neutrality indices for *An. stephensi* populations calculated from the nucleotide data set of the mitochondrial cytochrome c oxidase 1 (CO1) gene.

Population	N	S	Hap	Hapd ± VarHapd	Pi	TajimaD	FuFs
North Darfur	6	8	6	1.0 ± 0.00926	0.009	−0.6231	−3.178
River Nile	11	6	6	0.873 ± 0.00499	0.00617	0.1874	−1.375
Khartoum	5	4	4	0.9 ± 0.02592	0.00575	0.2734	−1.012
Red Sea	187	11	11	0.539 ± 0.00169	0.00268	−1.2208	−4.434
Kassala	94	11	11	0.579 ± 0.00339	0.00357	−1.1133	−3.962
El Gedarif	96	11	11	0.64 ± 0.00279	0.00523	−0.396	−1.963
Al Gezira	1	n.d.	1	n.d.	n.d.	n.d.	n.d.
India	27	2	3	0.274 ± 0.01143	0.00082	−0.9543	−1.052
Iran	2	0	1	n.d.	0	n.d.	n.d.
Pakistan	29	3	4	0.2 ± 0.00955	0.00059	−1.7326	−3.324 *
Saudi Arabia	8	3	4	0.643 ± 0.0339	0.00216	−1.4475	−1.832
Sri Lanka	18	1	2	0.471 ± 0.00678	0.00135	1.1662	1.215
United Arab Emirates	1	n.d.	1	n.d.	n.d.	n.d.	n.d.
Djibouti	1	n.d.	1	n.d.	n.d.	n.d.	n.d.
Ethiopia	1	n.d.	1	n.d.	n.d.	n.d.	n.d.
Sudan ^¥^	3	1	2	0.667 ± 0.09877	0.00192	n.d.	0.201

N: Number of sequences; S: number of segregating sites; Hap: number of haplotypes; Hapd ± VarHapd: haplotype diversity ± variance of haplotype diversity; Pi: nucleotides diversity; n.d.: not determined; ^¥^: indicates previously published sequences from Sudan; *: significance level < 0.05.

**Table 3:** Pairwise fixation index (Fst test values) between *An. stephensi* populations calculated from the nucleotide data set of CO1 gene.

Populations *	North Darfur	River Nile	Khartoum	Red Sea	Kassala	Gedarif	India	Iran	Pakistan	Saudi Arabia	Sri Lanka
River Nile	0.058	-	-	-	-	-	-	-	-	-	-
Khartoum	0.055	0.036	-	-	-	-	-	-	-	-	-
Red Sea	0.423	0.203	0.415	-	-	-	-	-	-	-	-
Kassala	0.394	0.171	0.372	0.004	-	-	-	-	-	-	-
El Gedarif	0.360	0.155	0.324	0.054	0.014	-	-	-	-	-	-
India	0.484	0.282	0.513	0.030	0.058	0.136	-	-	-	-	-
Iran	0.624	0.579	0.688	0.678	0.621	0.545	0.876	-	-	-	-
Pakistan	0.484	0.273	0.509	0.048	0.064	0.135	0.023	0.906	-	-	-
Saudi Arabia	0.652	0.672	0.682	0.796	0.745	0.641	0.879	0.927	0.885	-	-
Sri Lanka	0.485	0.304	0.512	0.065	0.106	0.168	0.126	0.824	0.224	0.863	-
Sudan	0.400	0.125	0.259	0.226	0.170	0.156	0.416	0.800	0.397	0.805	0.431

* Populations consisting of one sequence were not included in the comparison.

## Data Availability

All data generated during this study are included this article and the Supplementary Materials.

## Supplementary Materials

The following supporting information can be downloaded at: https://www.mdpi.com/article/10.3390/biology11030409/s1, Table S1: The accessions number of sequences of *An. stephensi* that are currently available in the GenBank and were included in the analysis and comparison of our sequences. Table S2: *p*-values of the pairwise Fst test was computed to estimate the degree of gene flow among the different populations. Table S3: Pairwise fixation index (Fst values) between *An. stephensi* populations calculated from the nucleotide data set of the mitochondrial cytochrome c oxidase subunit 1 (cox1) gene. Table S4: The distribution of the detected haplotypes among the different study sites compared to previously published *An. stephensi* cytochrome c oxidase 1 (CO1) sequences.
