# Supplementary material for: The Phylodynamic and Spread of the Invasive Asian Malaria Vectors, Anopheles stephensi, in Sudan
Source: Biology (Basel). 2022 Mar 7;11(3):409. doi: 10.3390/biology11030409 (PMC8945054; doi:10.3390/biology11030409)
Supplement: Supplementary file 1 [file biology-11-00409-s001.zip › biology-1587586-supplementary.pdf]

Supplementary File 1:

Table S1: The accessions number of sequences of *An. stephensi* that are currently available in the GenBank and were included in the analysis and comparing of our sequences.

India:

DQ154166.1	AY729980.1	DQ310143.1	DQ310148.1	DQ317594.1	MG571266.1
MH512893.1	MH538702.1	MH538703.1	MH538704.1	MH538705.1	MK265738.1
MK265740.1	MK265741.1	MK265742.1	MK265743.1	MK265744.1	MK704465.1
MK726121.1	LR736010.1	LR736014.1	LR736013.1	LR736015.1	MN329060.1
MN660044.1	MT899149.1	MW549046.1			

Pakistan:

KF406680.1	KF406682.1	KF406683.1	KF406684.1	KF406685.1	KF406686.1
KF406687.1	KF406688.1	KF406689.1	KF406690.1	KF406691.1	KF406692.1
KF406693.1	KF406694.1	KF406695.1	KF406696.1	KF406697.1	KF406698.1
KF406699.1	KF406700.1	KF406701.1	KF406702.1	KF406703.1	KF406704.1
KF406705.1	KF406706.1	KF406707.1	KF406708.1	KJ528895.1	

Sri Lanka:

MF124608.1	MF124609.1	MF124610.1	MF124611.1	MF975722.1	MF975723.1
MF975724.1	MF975725.1	MF975726.1	MF975727.1	MF975728.1	MF975729.1
MF975730.1	MF975731.1	MG970564.1	MG970566.1	MG970565.1	MG970567.1

Saudi Arabia:

KJ528887.1	KJ528888.1	KJ528889.1	KJ528890.1	KJ528891.1	KJ528892.1
KJ528893.1	KJ528894.1				

Sudan:

MW197099.1 MW197100.1 MW197101.1

Iran:

FJ210893.1 FJ210894.1

Djibouti:

KF933378.1

Ethiopia:

MH651000.1

United Arab Emirates:

MK170098.1

Supplementary File 2.

Table S2: P values of the pairwise Fst test was computed to estimate the degree of gene flow among the different populations.

	North Darfur	River Nile	Khartoum	Red Sea	Kassala	Al Gedarif	India	Iran	Pakistan	Saudi Arabia	Sri Lanka
**River Nile**	0.008	-	-	-	-	-	-	-	-	-	-
**Khartoum**	0.007	0.006	-	-	-	-	-	-	-	-	-
**Red_Sea**	0.010	0.006	0.007	-	-	-	-	-	-	-	-
**Kassala**	0.010	0.006	0.007	0.003	-	-	-	-	-	-	-
**Al Gedarif**	0.011	0.007	0.008	0.004	0.005	-	-	-	-	-	-
**India**	0.010	0.005	0.007	0.002	0.002	0.004	-	-	-	-	-
**Iran**	0.012	0.007	0.009	0.004	0.005	0.006	0.003	-	-	-	-
**Pakistan**	0.009	0.005	0.007	0.002	0.002	0.003	0.001	0.003	-	-	-
**Saudi Arabia**	0.016	0.013	0.013	0.012	0.011	0.010	0.012	0.015	0.012	-	-
**Sri Lanka**	0.010	0.005	0.007	0.002	0.003	0.004	0.001	0.004	0.001	0.013	-
**Sudan**	0.009	0.005	0.005	0.003	0.003	0.004	0.002	0.005	0.002	0.011	0.003

Supplementary File 3:

Table S3: Pairwise fixation index (Fst values) between *An. stephensi* populations calculated from the nucleotide data set of the mitochondrial cytochrome c oxidase subunit 1 (cox1) gene.

Populations*	India	Iran	Pakistan	Saudi Arabia	Sri Lanka
**Iran**	0.876	-	-	-	-
**Pakistan**	0.023	0.906	-	-	-
**Saudi Arabia**	0.879	0.927	0.885	-	-
**Sri Lanka**	0.126	0.824	0.224	0.863	-
**Sudan**	0.067	0.593	0.071	0.729	0.104

*All sequences representing Sudan were included in Sudan population. Populations consisted of one sequence were not included in the comparison.

Supplementary File 4:

Table S4: The distribution of the detected haplotypes among the different study sites compared to the previously published ***An. stephensi*** cytochrome c oxidase 1 (CO1) sequences.

**Populations**	**Hap 01**	**Hap 02**	**Hap 03**	**Hap 04**	**Hap 05**	**Hap 06**	**Hap 07**	**Hap 08**	**Hap 09**	**Hap 10**	**Hap 11**	**Hap 12**	**Hap 13**	**Hap 14**	**Hap 15**	**Hap 16**	**Hap 17**	**Hap 18**	**Hap 19**
**North Darfur**	1	0	0	0	0	0	0	0	0	0	0	1	1	1	1	1	0	0	0
**River Nile**	3	0	0	3	0	0	0	0	0	0	0	1	2	0	0	0	1	1	0
**Khartoum**	1	0	0	1	0	0	0	0	0	0	0	2	0	1	0	0	0	0	0
**Red Sea**	124	24	7	10	5	5	8	1	1	1	1	0	0	0	0	0	0	0	0
**Kassala**	60	8	6	6	4	2	3	1	1	2	1	0	0	0	0	0	0	0	0
**Gezira**	0	0	0	1	0	0	0	0	0	0	0	0	0	0	0	0	0	0	0
**Gedarif**	56	6	12	2	3	4	2	3	3	2	3	0	0	0	0	0	0	0	0
**Pakistan**	26	0	0	1	0	0	0	0	0	0	1	0	0	0	0	0	0	0	1
**Djibouti**	1	0	0	0	0	0	0	0	0	0	0	0	0	0	0	0	0	0	0
**Sri Lanka**	12	6	0	0	0	0	0	0	0	0	0	0	0	0	0	0	0	0	0
**India**	23	2	0	0	2	0	0	0	0	0	0	0	0	0	0	0	0	0	0
**Sudan***	1	0	0	2	0	0	0	0	0	0	0	0	0	0	0	0	0	0	0
**UAE**	1	0	0	0	0	0	0	0	0	0	0	0	0	0	0	0	0	0	0
**Ethiopia**	0	0	0	1	0	0	0	0	0	0	0	0	0	0	0	0	0	0	0
**Saudi Arabia**	0	0	5	0	0	0	0	1	1	1	0	0	0	0	0	0	0	0	0
**Iran**	0	0	0	0	0	2	0	0	0	0	0	0	0	0	0	0	0	0	0

Hap: haplotype, **UAE:** United Arab Emirates. **Sudan*:** indicates previously published sequences from Sudan.
